# Neuropsychiatric manifestations after gastric bypass progressing to status epilepticus resolving with pyridoxine: a case report and literature review

**DOI:** 10.1097/MS9.0000000000003406

**Published:** 2025-05-26

**Authors:** Siddharth Patel, Taylor Faust, Anil Chimakurthy, Prutha Pathak, Mc Anto Antony, Mrudula Thiriveedi, Sujatha Baddam

**Affiliations:** a Decatur Morgan Hospital, Decatur, AL, USA; b Alabama College of Osteopathic Medicine, Dothan, AL, USA; c North Alabama Medical Center, Florence, AL, USA; d Medical University of South Carolina/AnMed campus, Anderson, SC, USA; e Huntsville Hospital, Huntsville, AL, USA

**Keywords:** bariatric surgery, gastrectomy, pyridoxine, seizure, vitamin B6

## Abstract

**Introduction and Importance:**

Vitamin B6 (pyridoxine) deficiency is a rare but reversible cause of seizures in adults, which is often overlooked in post gastrectomy patients. This case highlights the critical role of nutritional supplementation in preventing severe complications such as neuropsychiatric manifestations and seizures, emphasizing the importance of vigilance in postoperative care and patient follow-up.

**Case presentation:**

A 46-year-old woman with a history of partial gastrectomy presented with intermittent confusion and somnolence for a duration of 6 months. Physical exam revealed temporal wasting, dry skin, and a low BMI. Laboratory tests showed undetectable levels of vitamin B6, while an electroencephalogram (EEG) demonstrated electrographic status epilepticus. Despite antiepileptic therapy the patient’s symptoms continued until high dose intravenous vitamin B6 was administered, leading to rapid improvement clinically and on EEG. The patient was discharged on oral pyridoxine and multivitamins, remaining seizure-free including at her 6-month follow-up

**Clinical discussion:**

This case illustrates the rare occurrence of adult-onset seizures due to vitamin B6 deficiency, following gastric surgery. A thorough literature review shows a link between pyridoxine deficiency and seizures, specifically in the context of malnutrition or chronic health conditions. This case underscores a need for routine preoperative and postoperative nutritional assessments, including micronutrient level monitoring in order to mitigate these risks.

**Conclusion:**

This case illustrates an extremely rare but preventable clinical syndrome of vitamin B6 deficiency and re-emphasizes the role of nutritional supplementation after gastrectomy. Prompt recognition and treatment of this condition are essential, highlighting the critical role of comprehensive nutritional care and postoperative management of gastrectomy patients.

**HIGHLIGHTS**

- Vitamin B complex deficiency can develop after gastrectomy if routine supplementation of vitamins is not taken. It can present with skin, eye, or hair manifestations. Very rarely confusion and seizures occur due to vitamin B6 deficiency.
- With an increasing number of gastric surgeries, understanding postoperative nutritional management becomes vital. We explore a rare entity of post-gastrectomy seizure responding to vitamin B6.
- Vitamin B6 deficiency could be considered as a cause of refractory seizures. We emphasize the importance of preoperative and postoperative nutritional assessment, including frequent assessments of vitamin levels to prevent deficiencies in gastric surgery patients.

## Introduction

Partial gastrectomy is commonly performed to treat various gastric conditions requiring resection such as peptic ulcer disease, benign gastric tumors, and traumatic injuries[1] . Total gastrectomy may be indicated for certain gastric malignancies, including adenocarcinoma, gastrointestinal stromal tumors, hemorrhagic gastritis unresponsive to medical or endoscopic management, etc[2]. In contrast, sleeve gastrectomy is performed to treat severe obesity. All forms of gastrectomy can increase the risk of micronutrient deficiencies, including the deficiency of vitamin B complex[3]. This may be due to poor preoperative nutritional status, reduced postoperative intake, impaired absorption, and inadequate supplementation.

Electrolyte imbalances, drugs, and toxins are recognized reversible causes of seizures. Vitamin B6 (pyridoxine) dependent epilepsy is a well-documented cause of neonatal seizures. However, its role in adult-onset epilepsy is rarely reported in the literature. We present a case of a young woman with this rare presentation, described according to the SCARE 2023 criteria[4] .

## Case report

A 46-year-old woman with a history of partial gastrectomy with Billroth-I anastomosis for non-healing gastric ulcers about 4 years prior, presented to the emergency department with intermittent altered mental status over 6 months. The patient reported episodes of drowsiness and confusion that improved with rest and sleep. These episodes, which lacked identifiable triggers, lasted for 2-4 hours and occurred 2-3 times weekly with gradually increasing frequency. She had recently been diagnosed with seizures based on electroencephalogram (EEG) findings following a prior hospitalization for similar symptoms. Her medical history included hyperlipidemia, hypothyroidism, chronic obstructive pulmonary disease, gastroesophageal reflux disease, peptic ulcer disease, erosive gastritis and antrectomy with vagotomy (performed approximately 5 years prior). She reported allergies to morphine and codeine. Medications included over the counter non-steroidal anti-inflammatory for chronic back pain and brivaracetam. However, she had not been taking the vitamins prescribed post-gastrectomy due to cost.

On examination, her vital signs were blood pressure of 122/66, heart rate of 84, respiratory rate of 14, O2 at 93%, temperature of 98.9°F, and BMI of 17.8 kilograms/meter[2]. She appeared drowsy but arousable, and was oriented to person and place. She was ill-appearing, looked older than her stated age, and had dry skin and frizzy hair. Pupils were equal and reactive; reflexes were symmetrical at 2+ with no motor or focal or sensory deficits.

**Figure 1.**
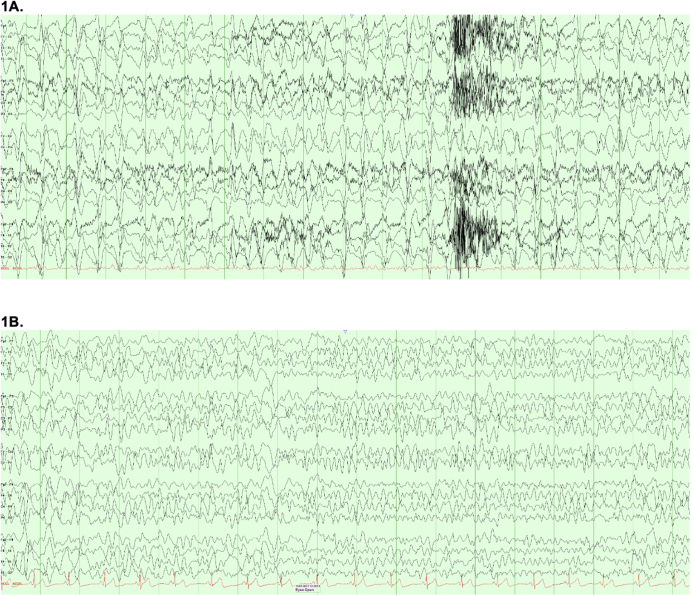
Electroencephalogram recordings throughout our patient’s disease history.

**Figure 2.**
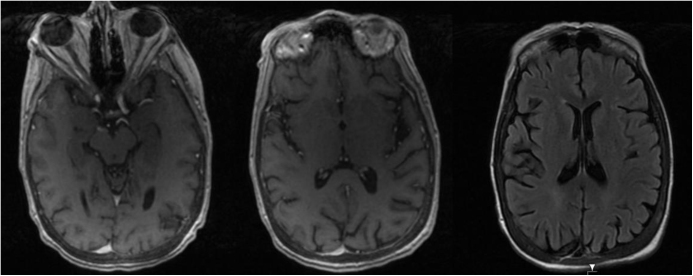
MRI brain with and without contrast – T1 weighted FSE images.

A complete blood count, comprehensive metabolic panel, urinalysis and serum vitamin levels were obtained (Table 1). A non-contrast head CT scan showed moderately advanced chronic microvascular ischemic changes. EEG demonstrated electrographic status epilepticus (Fig. 1A), with generalized, frequent sharp waves in the background of mild slowing. Despite the addition of IV valproate to brivaracetam, her clinical status did not improve. Vitamins B6 levels were found to be undetectable (<2 mcg/L). She was started on, 100.0 mg of high-dose IV vitamin B6 was taken daily for three days, followed by 50.0 mg PO twice daily. Her mental status improved significantly within 24 hours, and a follow-up EEG showed improvement with no epileptiform activity (Fig. 1B). She was discharged home on high dose oral 50.0 mg of vitamin B6 twice daily, a multivitamin, brivaracetam and divalproex. Further outpatient workup with lumbar puncture ruled out autoimmune and paraneoplastic encephalitis (Table 1). A brain MRI performed after discharge revealed no structural abnormalities (Fig. 2). A 6-month follow-up EEG confirmed continued seizure-free state.

**Table 1:** Laboratory investigations with autoimmune and paraneoplastic encephalitis workup

Initial blood tests	Lab values	Reference ranges
Hematology		
WBC	4.09 × 1000 U/L	(4.8–10.8 × 1000 U/L)
RBC	3.76 × MIL g/dL	(4.2–5.4 × MIL g/dL)
HgB	10.9 g/dL	(12.0–16.0 g/dL)
Hct	34.70%	(37.0–47.0%)
Coagulation		
PT	25.6 seconds	(11.0–16.0 seconds)
PTT	65.6 seconds	(22.3–41.8 seconds)
Chemistry		
Sodium	136 mmol/L	(136–145 mmol/L)
Potassium	3.6 mmol/L	(3.5–5.1 mmol/L)
Carbon dioxide	22 mmol/L	(25–35 mmol/L)
Creatinine	1.0 mg/dL	(0.5–0.9 mg/dL)
Total bilirubin	<0.15 mg/dL	(0.2–1.0 mg/dL)
AST	33 U/L	(10–30 U/L)
Alkaline phosphatase	220 U/L	(32–104 U/L)
C-reactive protein	17.83 pg/mL	(5–192 pg/mL)
Plasma Lactate	0.3 mmol/L	(0.5–2.2 mmol/L)
Vitamin B6	<2 mcg/L	(5–50 mcg/L)
Vitamin B12	1029 pg/mL	(243–894 pg/mL)
Folate	>20 ng/dL	>4.7 ng/dL
Magnesium	2.1 mg/dL	(1.5–2.7 mg/dL)
Urinalysis		
Urine protein	100 mg/dL	(Negative mg/dL)
Urine blood	Small	(Negative)
Serology		
RPR	Non-reactive	
**Test**	**Results**	**Reference**
Epilepsy, autoimmune/paraneoplastic panel, CSF		
AMPA-R Ab CBA, CSF	Negative	Negative
Amphiphysin Ab, CSF	Negative	Negative
AGNA-1, CSF	Negative	Negative
ANNA-1, CSF	Negative	Negative
ANNA-2, CSF	Negative	Negative
ANNA-3, CSF	Negative	Negative
CASPR2-IgG CBA, CSF	Negative	Negative
CRMP-5-IgG, CSF	Negative	Negative
DPPX Ab IFA, CSF	Negative	Negative
GABA-B-R Ab CBA, CSF	Negative	Negative
GAD-65 Ab Assay, CSF	0.00	nmol/L ⇐0.02
GFAP IFA, CSF	Negative	Negative
LGI1-IgG, CSF	Negative	Negative
mGluR1 Ab IFA, CSF	Negative	Negative
Neurochondrin IFA, CSF	Negative	Negative
NMDA-R Ab CBA, CSF	Negative	Negative
PCA-Tr, CSF	Negative	Negative
PCA-2, CSF	Negative	Negative

## Literature review and results

To understand seizures in adults with severe protein calorie malnutrition, and its association with vitamin B6 deficiency, we performed a thorough literature search using PubMed. Our keywords included “vitamin B6,” “pyridoxine,” “seizures,” and “adults.” After reviewing titles of 180 results, we excluded articles that did not clearly mention seizure and vitamin B6. In addition, we excluded animal studies and articles that included the pediatric population or talked about toxicity related to drugs or other metabolic derangements. This eventually resulted in 27 articles for abstract review. After a detailed review of selected abstracts, ultimately 8 case reports relevant to our research question were included (Table 2). We used only one search engine for thorough literature search since there was a scarcity of literature directly addressing our research question, particularly those meeting the specific criteria outlined earlier.

**Table 2:** Cases of seizures associated with vitamin B6 deficiency

Article title	Authors	Coexisting comorbidities	Response to vitamin B6 supplementation
Vitamin B6 deficiency: a potential cause of refractory seizures in adults	Gerlach AT, *et al*	Alcohol-induced or hepatitis C-associated liver disease	Seizure ceased: after IV then oral pyridoxine supplementation
Seizures related to vitamin B6 deficiency in adults	Lee DG, *et al*	10 year history of heavy alcohol use	Seizure ceased: following nutritional vitamin B6 supplementation
Diagnosis of pyridoxine-dependent epilepsy in an adult presenting with recurrent status epilepticus	Osman C, *et al*	Two pathogenic ALDH7A1 mutations	Seizures ceased: after starting oral pyridoxine supplementation
Seizures caused by pyridoxine (vitamin B6) deficiency in adults: a case report and literature review	Tong Y.	Parkinson’s disease, stage 4 chronic renal failure, recent pelvic fracture due to fall	Seizures ceased: after oral pyridoxine vitamin supplementation
Epileptic status refractory to conventional treatment caused by vitamin B6 deficiency	Valle-Morales L, *et al*	29th week of pregnancy	Seizures ceased: after IV pyridoxine
Status epilepticus after gastric bypass surgery	Torcida Sedano N, *et al*	Recent gastric bypass surgery	Seizures ceased: after nutrition and vitamin pyridoxine supplementation
From mild gait difficulties to a sudden coma: a rare case of Marchiafava–Bignami disease	De Ryck H, *et al*	Hyperglycemia, Marchiafava–Bignami disease, Epstein–Barr virus hepatitis, EBV positive diffuse large B-cell lymphoma, diabetes mellitus	Seizures ceased: after high-dose IV pyridoxine
Case of an 81-year-old woman with theophylline-associated seizures followed by partial seizures due to vitamin B6 deficiency	Kuwahara H, *et al*	Chronic bronchitis	Seizures ceased: after IV pyridoxine

## Discussion

Presented here is a compelling case of a young woman who developed severe malnutrition following gastrectomy due to non-compliance with micronutrient supplements. She experienced neuropsychiatric symptoms and ultimately presented with seizures. Vitamin B6 deficiency was not initially considered, as it is an uncommon cause of neuropsychiatric manifestations such as seizures. It was not high on our initial differential diagnosis, and more common reversible causes including electrolyte abnormalities, infections, and cerebrovascular events were ruled out first through blood investigations, lumbar puncture, and brain MRI. Notably, she had a severe vitamin B6 deficiency, and her seizures responded to IV vitamin B6 administration. This case highlights the importance of post-gastrectomy nutrition and indicates the need for further investigation into the role of vitamin B6 in adult-onset seizures.

Both macro- and micronutrient deficiencies can develop after gastrectomy; sometimes years postoperatively[5]. Deficiencies in fat-soluble vitamins (A, D, E, and K), water soluble vitamins (B complex and C), and trace minerals (iron, zinc, selenium, copper, and calcium) have been well-documented following bariatric surgeries. While vitamin B12 deficiency is more common, vitamin B6 deficiency has also been described after gastrectomy.[6] Iron and folate deficiency can cause anemia; and vitamin B12 deficiency can have neuropathic manifestations^[7,8]^. Vitamin B1, B2, B3, and B6 deficiencies can result in a variety of dermatological, constitutional, and even neurological symptoms post-gastrectomy^[9-12]^.

**Figure 3.**
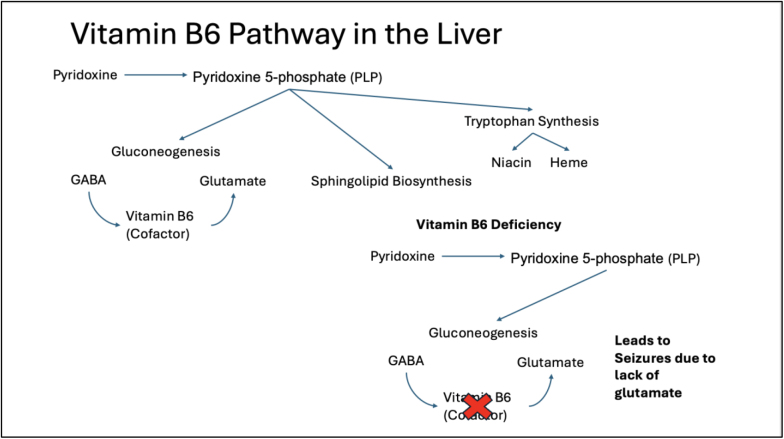
Vitamin B6 pathway and vitamin B6 deficiency pathway leading to seizures.

Vitamin B6 is a water-soluble vitamin found in meat, fish, nuts, beans, grains, fruits, and vegetables[13]. It is absorbed in the jejunum and metabolized in the liver to its active form, pyridoxine 5-phosphate (PLP) in the liver[14]. It plays a critical role in gluconeogenesis, sphingolipid biosynthesis, conversion of tryptophan to niacin, and the synthesis of heme and neurotransmitters[15] (Fig. 3). Though dietary deficiency is rare, secondary vitamin B6 may occur due to its impaired metabolism from drugs like isoniazid and hydralazine[16]. Other causes include protein-calorie malnutrition, malabsorption, excessive alcohol use, Hodgkin lymphoma, and sickle cell anemia[17]. Mild deficiency can be asymptomatic for years 9,18, while moderate deficiency may present with stomatitis, glossitis, seborrheic dermatitis and cheilosis, and occasionally neuropsychiatric symptoms like irritability, confusion, and depression 9,18. In its most severe form, it can lead to microcytic anemia and seizures^[9,18]^. Pyridoxine dependent epilepsy is well known in neonates with genetic defects in vitamin B6 metabolism[19], but its occurrence in adults due to secondary pyridoxine deficiency is exceedingly rare.

Vitamin B6 plays a vital role in the nervous, immune, and endocrine systems. PLP serves as a cofactor in synthesizing gamma-aminobutyric acid (GABA) from glutamate^[20-22]^ and its deficiency can lower the seizure threshold[23]. In the case of our patient, intermittent confusion and somnolence were likely due to suspected seizures. Despite broad-spectrum anti-epileptic medication, symptoms persisted. It was not until vitamin B6 levels were correct with IV supplementation, her symptoms resolved and it led to rapid clinical improvement.

Gerlach *et al*[24] reported three adult patients with refractory seizures that resolved with vitamin B6 supplementation. Lee *et al*, Osman *et al*, Tong *et al*, and Valle-Morales *et al* have also described similar cases of vitamin B6 deficiency associated with epilepsy in adults^[20,25-27]^. Sedano *et al* described a 39-year-old female presenting with status epilepticus after gastric bypass attributed to multiple nutrient and vitamin deficiencies^[26,28]^. De Ryck *et al* reported multiple generalized epileptic seizures in a 66-year-old man with Marchiafava–Bignami disease and nutritional deficiency including that of vitamin B6[29]. Kuwahara *et al* published a case involving an 81-year-old female who after theophylline-associated seizures developed partial seizures due to vitamin B6 deficiency[30].

All these cases document seizures in the context of vitamin B6 deficiency, frequently with underlying malnutrition or chronic illness. However, only one involved gastrectomy as the underlying mechanism for malnutrition, as in our case[24]. While establishing causality is challenging, the association between high dose vitamin B6 administration and clinical improvement in our patient supports a potential link. The existing literature reinforces our hypothesis and highlights the need for exploring this further. Given the prevalence of B complex vitamin deficiencies after gastrectomy, this case reaffirms the importance of only routine supplementation and ongoing micronutrient monitoring in post-surgical patients. One significant limitation in this patient’s case was the inability to afford prescribed vitamins, which likely contributed to her seizure activity. This financial barrier, compounded by limited access to healthcare and inadequate follow-up of vitamin levels post-gastrectomy, may have been a leading factor in her condition. Further clinical research is needed to better understand the complications associated with bariatric surgeries. This would not only strengthen the evidence presented in this case but also support improved prevention, management, and intervention strategies for similar patients.

## Conclusion

With the rising prevalence of gastric surgeries, effective postoperative nutritional management is increasingly essential for patients. Vitamin B6 deficiency can remain underdiagnosed until it presents with severe manifestations, as seen in our patient. We describe a rare presentation of post-gastrectomy seizures that responded to vitamin B6 supplementation. Although establishing a definitive causal association is challenging, our review of the literature suggests that vitamin B6 deficiency should be considered in the cases of refractory seizures. This case highlights the importance of comprehensive nutritional assessment both pre and postoperatively, including frequent monitoring of vitamin levels to help prevent deficiencies.

## Data Availability

Not applicable since this is a single case report. None of the contents of the manuscript is derived from any publicly available dataset.
